# The COVID-19 Pandemic – A Diagnostic Industry Perspective

**DOI:** 10.3389/fcimb.2022.862440

**Published:** 2022-03-24

**Authors:** Shubhagata Das, Sherry Dunbar

**Affiliations:** Luminex Corporation, A DiaSorin Company, Austin, TX, United States

**Keywords:** COVID-19, diagnostic industry, diagnostic testing, assay development, diagnostics manufacturer

## Abstract

The COVID-19 pandemic has brought about unprecedented changes to all facets of the healthcare system, including the diagnostic industry. The pandemic has highlighted both the challenges and strengths of the industry and has also provided valuable insights on how to be better prepared for future pandemics. In this perspective article, we describe the challenges faced by the diagnostic industry in general, particularly the difficulties encountered by Luminex Corporation, a diagnostic assay development and manufacturing company located in Austin, Texas, USA, as well as the mitigation strategies employed. In addition to discussion of the key challenges, the article provides insights on the lessons learned and steps that can be undertaken to better prepare for future outbreaks.

## Introduction

The coronavirus-19 (COVID-19) pandemic has posed unprecedented global challenges to a multitude of sectors and industries. The pandemic not only overburdened the healthcare system but it has also significantly affected the in-vitro diagnostic industry. During the early days of the pandemic, hospitals and emergency centers were overpopulated with patients, resulting in an insufficient number of available beds and personnel, as well as an immense upsurge in need for diagnostic testing kits for identifying the SARS-CoV-2 virus, which were not yet readily available (Centers for Disease Control and Prevention, 2020). Rapid diagnosis and testing was paramount for allocating hospital resources, appropriate patient cohorting, administering effective therapeutic measures, and implementing adequate quarantining procedures (Rosenthal, 2020). Manufacturers of diagnostic assays witnessed a rapid escalation in their research and development efforts to develop tests for SARS-CoV-2. All diagnostic companies worked towards a common goal – bringing about a highly sensitive and specific test rapidly in the market and making it accessible to clinical laboratories to meet patient needs and guide the isolation practices for potentially infectious individuals. However, due to the unprecedented nature of the pandemic, the industry overall encountered tremendous challenges in terms of the available workforce, production capacity, and ongoing supply chain issues. Additionally, the constant change in the available information and guidance from regulatory agencies created further confusion that required constant communication and monitoring of the ongoing pandemic.

Luminex^®^ Corporation is a diagnostic assay development and manufacturing company located in Austin, Texas that offers a wide range of products for clinical diagnostics and biomedical research. The company offers both targeted and syndromic molecular testing panels for different disease states. Additionally, using Luminex’s xMAP^®^ Technology, users can perform a wide range of protein- and nucleic acid-based multiplex assays, which can simultaneously detect up to 500 targets in a single reaction. In this perspective article, we describe the unique challenges faced by the diagnostic industry during the COVID-19 pandemic, particularly focusing on the difficulties encountered by Luminex, and discuss the possible strategies that can be implemented to tackle future pandemics.

## Challenges in Research And Development

During the COVID-19 pandemic, rapid and accurate diagnosis of the causative pathogen in both symptomatic and asymptomatic patients was particularly critical, as it supported appropriate patient cohorting, quarantine duration, and subsequent therapy and treatment (Rosenthal, 2020). On January 10, 2020, the viral genome sequence for SARS-CoV-2 was released for immediate public health support, and since then, hundreds of diagnostic assays have been developed commercially for the rapid detection of the novel coronavirus (Wuhan-Hu-1, GenBank accession number MN908947). From an industry perspective, developing, validating, manufacturing, and finally commercializing a diagnostic assay is an elaborate and complex process that requires interdepartmental collaboration, detailed planning, and effective time management. Several critical steps are involved in this process, including defining the need for the product, determining the clinical utility, and establishing the performance criteria of the diagnostic assay.

Similar to other diagnostic companies, Luminex offers multiple solutions for several disease states based on the needs of the clinical diagnostic laboratories. For any type of disease, the diagnostic test can be a sample-to-answer low-plex assay that may detect a few targets or a high-complexity multiplex assay that can detect many targets from a single sample. Additionally, laboratories can use extraction cassettes to design and validate their own laboratory-developed assays for clinical diagnostic purposes. Therefore, with a sudden surge in the need for SARS-CoV-2 diagnostic tests, it was essential for management and R&D to prioritize the assay platforms and chemistries for developing a SARS-CoV-2 assay based on the needs of clinical laboratories and patients. The company needed to decide whether they should prioritize the development of a molecular RT-PCR-based assay for detecting the current infection or a serological assay for determining prior exposure to the pathogen or possible immunity, or both. In addition to deciding on the type of chemistry and assay, the R&D scientists also had to quickly develop the primer and probe designs and determine the relevant sample types and transport system/media to include in the assay claims.

The SARS-CoV-2 virus was a novel coronavirus, and therefore scientists all over the world had no access to pre-existing data regarding the pathogen. Globally, numerous studies were conducted by researchers to understand the viral structure, its pathogenicity, and transmissibility (Hsu et al., 2020). SARS-CoV-2-related available information was evolving rapidly and with the emergence of new variants and mutations, which was challenging for the R&D teams, as they had to consistently re-evaluate the developed assays to incorporate new mutations as needed. Conflicting information regarding the utility of certain assays, such as the SARS-CoV-2 serological or neutralizing assay, also caused a significant strain on research and development efforts during the early days of the pandemic. During this time, one of the primary activities of the R&D team was staying up-to-date on the constantly changing information and revising their efforts accordingly.

## Challenges in Manufacturing and Supply Chain

The availability of biological materials, reagents, and other laboratory accessories is paramount for developing and commercializing any diagnostic assay. During the pandemic, laboratories and manufacturers were developing and running assays at a higher than usual pace, leading to a critical shortage of basic and essential laboratory equipment such as pipette tips, PCR reagents, tubes, and personal protective equipment (PPE), including gloves and masks. Additionally, with the World Health Organization (WHO) urging countries to ramp up testing for COVID-19, commercial assay manufacturers (including Luminex) had to surge the production of their COVID-19 assay kits which caused tremendous pressure on suppliers for sourcing assay manufacturing components such as plastics, molds, and other raw materials (World Health Organization, 2020). Most of the suppliers were not classified strictly as a healthcare business, and they were forced to shut down during the pandemic even though they were supplying critical components required for developing assay kits.

With such a massive upscale in production, another aspect to monitor and control was the quality of the assay kits being manufactured. The manufacturing team had to run a more vigorous and stringent quality control process as more kits were being produced than usual. A higher rate of manufacturing also requires adjusting the logistical chain to ensure around-the-clock production, shipping, and delivery of the testing kits. There was a tremendous demand for skilled labor and technicians, as Luminex was trying to accommodate three 8-hour manufacturing shifts in a day to meet the production demands from the customers. There were massive turnovers as demand for skilled manufacturing technicians was high, which required a constant chain of hiring and training of new personnel. The manufacturing and supply chain teams also had to work around closed borders, canceled flights, delivery delays, and limited suppliers’ stocks. Overall, the primary challenge for the team was to obtain and source the required raw material on time, produce enough kits to meet the demand, ensure the quality of the manufactured kits, and finally ship them to their destinations in a timely manner.

The high demand for testing kits also posed a significant financial burden on industries. In addition to obtaining more supplies upfront and hiring more personnel, companies also needed to invest in capital equipment to meet the production demands. At Luminex, the manufacturing team installed new automation components to speed up the processes, which required additional investment and training. Moreover, as the other instruments were working at a higher capacity than usual, it escalated the failure rates of modules and other components that demanded an increase in routine maintenance services.

## Challenges in Regulatory Affairs

The Food and Drug Administration’s (FDA) clearance of any medical devices and diagnostic assays is critical to obtain before product commercialization in the United States. It ensures that the device or test meets the required performance, safety, and effectiveness standards. Under normal circumstances, the Luminex regulatory affairs team works for months to obtain the necessary data required for FDA IVD-clearance by conducting rigorous clinical trials using prospective and retrospective patient samples. The approval process further requires detailed documentation of the performed clinical trial, and often takes months post-data submission to obtain the final clearance for diagnostic use. However, during emergencies, the FDA can clear diagnostic tests and devices under Emergency Use Authorization (EUA) after receiving the minimum required data or evidence regarding the safety and efficacy of the product (U.S. Food and Drug Administration, 2022).

During the COVID-19 pandemic, the FDA started issuing EUAs for SARS-CoV-2 diagnostic kits to meet the required testing needs for initiating appropriate patient isolation protocols and therapy. Although the EUA guidelines were less stringent, validation and performance evaluation using either real or contrived clinical samples is still required to obtain the FDA clearance before commercialization of the assays. Getting clinical samples from patients was challenging during the initial days of the pandemic because of their high demand from various laboratories and manufacturers, which threatened industrial assay developers with significant delays for commercialization. At Luminex, the assay development and regulatory teams had to rely on contrived samples for assay development and initial testing, and *in silico* analyses were also performed for generating the required evidence and data. During the approval process, constant communication with the FDA was essential, as reviews and suggestions for required changes were obtained almost daily, which otherwise would typically take days or months. It was further challenging to meet the FDA EUA regulatory guidelines, as in some cases there was a need to modify requirements based on the progress on the pandemic and availability of new data.

## Challenges in Marketing and Sales

The marketing team plays a crucial role in any product launch and development in the diagnostic industry. Under the usual business regimen, the marketing department takes part in deciding the characteristics of the diagnostic assay and determines the target market segment, price points, and the overall positioning and messaging for the product. These things needed to be handled differently with the pandemic, as there was no time for detailed and meticulous planning. The primary focus of diagnostic companies was how to provide a faster and more accurate test to all of their customers, irrespective of market segmentation, to meet patient needs and help control the pandemic. A strategic plan for commercialization that may take several months had to be implemented in a few weeks.

The seriousness of the pandemic demanded a quicker assay development timeline and a rapid product launch using limited available resources. This incurred a sudden surge of a financial burden on companies from various standpoints such as research and development, manufacturing, and shipping. Similar to other diagnostic companies, Luminex had to seek out federal government aid and apply for funding to support their research and developmental activities. The Luminex team was able to secure several millions of dollars in funding from the U.S. Department of Health and Human Services’ Biomedical Advanced Research and Development Authority (BARDA) to support the development and validation of several projects, including a COVID-19 multiplex antibody test and a multiplex respiratory panel for Flu A/B, respiratory syncytial virus (RSV), and SARS-CoV-2 targets (Cision PR Newswire, 2022). Furthermore, due to the successful launch of the sample-to-answer SARS-COV-2 molecular assay, the company was able to secure additional funding for further improvements to manufacturing and alleviating supply chain issues.

The sales teams of the diagnostic companies also played a critical part during the pandemic. They had to prioritize their customers, meet their testing needs quickly, and provide adequate training and troubleshooting support. Since Luminex’s sample-to-answer PCR system also supports laboratory-developed tests (LDTs), many laboratories were developing their own assays using the sequences provided by the U.S. CDC and Wuhan Institute of Virology. Therefore, in addition to providing customers with the commercially developed assay, the team also had to take care of the high complexity labs that were using the general purpose reagents and consumables with their own primers to obtain their own EUA. This challenge was global as the company had to support laboratories worldwide.

Another significant challenge faced by the company was the rapidly changing information available during the initial days of the pandemic. Laboratories implemented multiple testing platforms to accommodate the changing requirements, high patient volume, and supply chain issues, which required a faster training time and a more demanding validation support for the assays they were using. The manufacturers had to stay on top of changing recommendations and guidelines which occurred as the pandemic evolved, and constant communication with the FDA and the CDC was vital during this time.

## Lessons Learned for Future

The COVID-19 pandemic brought about a significant change in the in-vitro diagnostic industry. While the pandemic brought the laboratories and industry together to focus on a common goal, it has also highlighted numerous challenges that may be encountered during the development and commercialization of a diagnostic test under emergency and critical circumstances. The diagnostic industry must learn from this experience and implement necessary steps to ensure better preparedness for such future emergencies.

A glaring shortcoming that became evident during this pandemic was the global shortage of essential raw materials required for developing an assay. Companies faced a shortage of reagents, PPE, and other necessary laboratory equipment, such as pipette tips. For the future, companies must evaluate and implement multi-sourcing and multi-manufacturing models to ensure that critical raw materials are always available. Companies need to identify materials and products that are absolutely critical for their businesses and have a reasonable stockpile inventory of those products to ensure they are always available. It is important to understand the supplier(s), their capacity, and maintain an overall healthy working relationship. Better internal surveillance of the epidemiological data is also needed in the diagnostic industry so that they can rationally predict an outbreak and can be prepared.

Additionally, IVD industry stakeholders, including laboratories, manufacturers, and government agencies, need to communicate amongst themselves to coordinate and cooperate in developing a comprehensive national response plan for future outbreaks. Collaborative strategies should be developed to ensure that testing supplies (reagents and consumables) would be available during the different stages of a pandemic. For future preparedness, administrative bodies should also collaborate with IVD assay manufacturers and provide funding to overstock their testing supplies such as reagents, PPE, and laboratory equipment, so an extra amount of necessary test supplies is readily available (O'Connor, 2021). However, manufacturers should consider if these supplies can be used or sold before expiration.

Diagnostic companies should identify their bottlenecks and capacity trigger points from a manufacturing standpoint. Manufacturers should clearly understand their production capacity and the steps needed to escalate it on demand. It is essential that production facilities have emergency escalation plans that would aid in rapid decision-making and expansion during emergencies. Communication within different departments is also vital in establishing goals and preparing for the future.

Another critical gap was the lack of a clear research and development response strategy, which includes better preparedness for developing an assay and collaborative efforts between different industries and stakeholders such as funding and regulatory agencies, government entities, epidemiological institutions, and researchers. Partnership efforts with different laboratories and contract research organizations for assay development can also be helpful to draw expertise from diverse areas such as biochemistry, virology, molecular biology, etc. that might assist with optimizing assay parameters. The inconsistencies observed in the diagnostic performance between different assays highlighted the need for a close partnership between assay manufacturers and component and material providers to reduce the risk of choosing suboptimal parts, specifically in sectors dealing with lateral flow based assays (Abate, 2020). Additionally, having a close relationship with clinical sample banks, government agencies, and hospitals is critical for obtaining clinical samples during emergencies for assay development and testing.

The COVID-19 pandemic has further demonstrated how regulatory timelines can be drastically improved and accelerated, and how assay developers can adapt and benefit from the accelerated pace. During an emergency, it is essential to determine the amount of oversight needed so that inadequate monitoring does not lead to inaccurate or faulty devices flooding the market or an over-extensive review creates a delay in test availability. Additionally, it is vital to establish clinical endpoints and desired performance parameters early to provide adequate transparency to the diagnostic assay developers. Regulatory agencies and policymakers should ensure that the diagnostic community can always access the required data infrastructure to evaluate different testing strategies (Wiegmann and Roca, 2021). Regulatory coordination is also of paramount importance on a global scale, and both national and international organizations should collaborate to develop comprehensive response plans and determine adequate regulatory approval processes. Evidence-based policymaking is essential during times of crisis as information changes quickly.

## Conclusion

The COVID-19 pandemic served as a revelation for the diagnostic industry, highlighting both its strengths and weaknesses. On the one hand, there were innovations, rapid responses towards fulfilling a common goal, and immense adaptability, whereas, on the other hand, there were massive manufacturing and supply chain issues, the inability to meet the required demand, and discrepancies in the knowledge disseminated from various sources. The three critical needs identified during the pandemic were timeliness, accuracy, and availability. The adaptations and innovations that have been embraced during this pandemic will surely cause a drastic change in the overall operations and functions of the diagnostic industry. Similar to other companies, Luminex is also identifying their shortcomings and the potential bottlenecks from every departmental standpoint and are implementing better operational processes for the future. The novel circumstances associated with the pandemic have facilitated an overall transformation and evolved the diagnostics industry towards more forward-facing patient-centric solutions that will endure even after the pandemic.

## Data Availability Statement

The original contributions presented in the study are included in the article/supplementary material. Further inquiries can be directed to the corresponding author.

## Author Contributions

Both authors contributed to the article and approved the submitted version.

## Conflict of Interest

The authors are employees of Luminex Corporation, A DiaSorin Company.

## Publisher’s Note

All claims expressed in this article are solely those of the authors and do not necessarily represent those of their affiliated organizations, or those of the publisher, the editors and the reviewers. Any product that may be evaluated in this article, or claim that may be made by its manufacturer, is not guaranteed or endorsed by the publisher.
